# The role of health perception in preventive care

**DOI:** 10.1016/j.apjon.2025.100689

**Published:** 2025-03-22

**Authors:** Negin Dorri

**Affiliations:** Department of Psychology and Educational Sciences, University of Tehran, Tehran, Iran

Scientific literature across disciplines consistently demonstrates that modifiable lifestyle risk factors—such as physical inactivity, poor nutrition, tobacco use, and excessive alcohol consumption—are critical determinants in the development of chronic diseases such as cardiovascular disease, cancer, and diabetes.^1^ Characterized by complex and often synergistic interactions, these factors contribute to disease onset and progression, potentially undermining treatment efficacy and shaping responses to medical interventions.

Preventive measures, such as adopting healthier lifestyles, have been shown to reduce risk and improve clinical outcomes.^2^ However, behaviors performed over extended periods become integral to an individual's daily routine and identity, making them resistant to change. Consequently, modifying health-related behaviors and influencing decision-making often necessitates long-term, tailored, and targeted interventions.

Perception, influenced by personal experiences, cultural contexts, and social influences, is a key determinant in the success of preventive efforts across primary, secondary, and tertiary care. Traditional models, such as the Health Belief Model—a cornerstone in health behavior research—underscore the pivotal role of perception in decision-making. This suggests that individuals are more likely to engage in preventive actions if they perceive themselves at risk and view the benefits as outweighing the risks. Therefore, individuals require time to process and internalize health information before adopting preventive measures and engaging in cognitive appraisal to assess risks and benefits. This process shapes their perception of vulnerability and readiness for behavior change.^3^ However, despite the well-established role of perception in behavior change, prevention measures often prioritize knowledge as the primary driver without fully accounting for how individuals interpret and respond to perceived risks.

Integrating perception into preventive care requires a shift toward tailored communication that considers prevailing beliefs, personal experience, and cultural background to bridge the gap between knowledge and action. Addressing these factors is crucial. As shown in Fig. 1, health perception interacts with barriers, facilitators, and lifestyle factors, shaping the success of preventive interventions towards behavioral change.

**Fig. 1:**
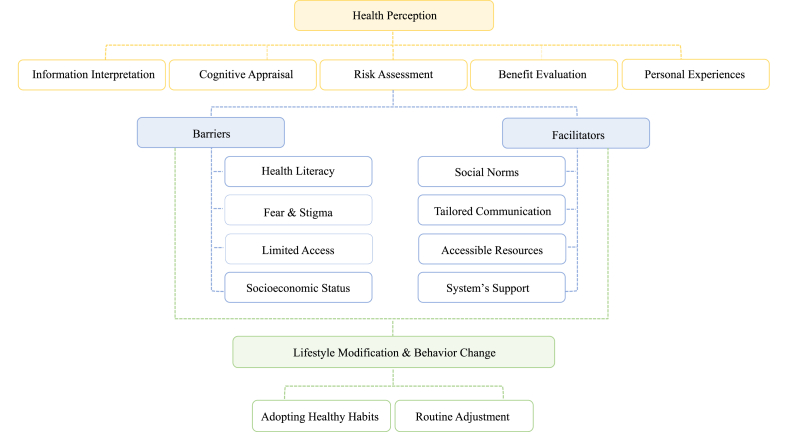
The role of health perception in shaping behavioral change and lifestyle modification.

In the context of cancer prevention, perception is not just a passive process but a dynamic force that shapes behaviors across the prevention continuum. Understanding how individuals perceive their susceptibility to cancer—whether as a predetermined genetic fate or a modifiable risk influenced by lifestyle—is crucial. It directly affects their willingness to adopt protective measures in primary prevention. Risky behaviors such as smoking, poor diet, and physical inactivity often persist when individuals do not perceive immediate consequences, leading them to underestimate long-term risks. This is particularly evident in sun exposure habits, where individuals who do not visually perceive the harm caused by ultraviolet (UV) radiation may dismiss skin cancer warnings as abstract or irrelevant. However, when UV imaging reveals hidden skin damage, it alters perception by making the risk tangible, evoking stronger emotional responses, and ultimately increasing the likelihood of protective behaviors such as sunscreen use.^4^

In secondary prevention, perceptual factors such as fear of a diagnosis or misinterpretation of symptoms can extend the patient interval, leading to delays in essential screenings like mammograms or colonoscopies.^5^ Similarly, in tertiary prevention, treatment adherence is often shaped by patients’ perceptions of its efficacy and concerns about adverse physical and non-physical side effects, possibly contributing to discontinuation.^6^ Addressing a select range of perceptual barriers, as highlighted in Table 1, is essential for bridging the gap between awareness and action across the different stages of prevention.

**Table 1:** Perception's influence across preventive stages.

Preventive Stage	Perception's Influence	Description
Primary prevention	Perceived susceptibility	Beliefs about whether cancer is a genetic fate or influenced by lifestyle affect willingness to adopt preventive behaviors.
	Perceived severity	Underestimating the seriousness of cancer can lead to dismissing prevention efforts, as the threat feels distant or non-urgent.
	Immediate vs. Long-Term consequences	Risky behaviors (e.g., smoking, poor diet) persist when individuals do not perceive immediate harm, leading to neglect of preventive measures.
	Efficacy of prevention measures	Skepticism about the effectiveness of preventive actions (e.g., exercise, healthy eating) can reduce motivation to engage in these behaviors.
	Immediate feedback	Visible outcomes (like sunburn) prompt immediate action, reinforcing the value of preventive measures compared to abstract, long-term risks.
Secondary prevention	Fear of diagnosis	Anxiety about cancer diagnosis may cause avoidance of screening tests, leading to delayed early detection.
	Underestimation of risk or symptoms	Misinterpretation or underestimation of symptoms may lead to complacency and delay seeking medical advice.
	Perceived difficulty of screenings	If procedures (e.g., colonoscopies) are seen as uncomfortable or invasive, individuals may postpone or skip them.
Tertiary prevention	Perception of treatment effectiveness	Doubts about treatment effectiveness (e.g., chemotherapy) may discourage patients from continuing treatment.
	Side effect fears	Concerns about severe side effects (e.g., fatigue, hair loss) may cause patients to discontinue or avoid treatment.
	Social support	Positive reinforcement from family or support groups shapes the individual's perception of their ability to successfully undergo treatment, fostering confidence and adherence, while negative social cues may undermine treatment commitment.

As the significance of lifestyle risk factors continues to grow in modern medicine, integrating perception-driven approaches into preventive care offers a critical path for improving health outcomes. Addressing perceptual barriers—whether rooted in fear, skepticism, or cultural beliefs—requires a multidisciplinary effort among researchers, clinicians, and policymakers. Health care professionals are crucial in aligning communication strategies with individuals’ experiences and beliefs, empowering them to move beyond awareness to foster lasting behavior change. Ultimately, transforming perceptions alongside education and health literacy will enable a future where preventive measures are understood, actively adopted, and maintained.

## Declaration of generative AI and AI-assisted technologies in the writing process

During the preparation of this work, the author used Grammarly software to check for grammatical accuracy and presentation. After using this tool, the author reviewed and edited the content as needed and takes full responsibility for the content of the publication.

## Funding

This study received no external funding.

## Declaration of competing interest

The author declare no conflict of interest.
